# Phytochemical and Pharmacological Properties of *Capparis spinosa* as a Medicinal Plant

**DOI:** 10.3390/nu10020116

**Published:** 2018-01-24

**Authors:** Hongxia Zhang, Zheng Feei Ma

**Affiliations:** 1 Department of Food Science, University of Otago, Dunedin 9054, New Zealand; 2 Department of Public Health, Xi’an Jiaotong-Liverpool University, Suzhou 215123, China

**Keywords:** *Capparis spinosa*, caper, cardiovascular disease, traditional medicine, flavonoids, rutin

## Abstract

Over the past decades, there has been increasing attention on polyphenol-rich foods including fruits and vegetables on human health. Polyphenols have been shown to possess some potential beneficial effects on human health and they are widely found in foods consumed by populations worldwide. *Capparis spinosa* (*C. spinosa*) is an important source of different secondary metabolites of interest to humankind. The traditional therapeutic applications of *C. spinosa* have been reported in Ancient Romans. Numerous bioactive phytochemical constituents have been isolated and identified from different parts (aerial parts, roots and seeds) of *C. spinosa* which are responsible alone or in combination for its various pharmacological activities. Therefore, this paper is a review of publications on the phytochemical and pharmacological properties of *C. spinosa*. There is insufficient evidence to suggest that *C. spinosa* or its extracts are able to improve the biomarkers of cardiovascular disease and diabetes. However, these studies used different parts of *C. spinosa* plant, methods of preparation and types of solvents, which cause the evaluation of activity of *C. spinosa* difficult and involve quite heterogeneous data. There is also evidence, although limited, to suggest benefits of *C. spinosa* in improving human health. Therefore, the relationship between *C. spinosa* and improved human health outcomes requires further study.

## 1. Introduction

Medicinal plants have been used since ancient times as therapeutic agents for the management of health and treatment of diseases because they possess health-promoting effects and contain bioactive components [1]. According to the World Health Organization (WHO) [2], 80% of the world’s populations rely mainly on traditional medicine. In China, 30–50% of the overall medicinal consumption is estimated from the preparations of traditional medicine [3]. Approximately 90% of the population in Germany reported that they have used natural remedies for certain health purposes [2]. Therefore, there is increasing use and popularity of traditional medicine in both the developing and industrialized countries [4], demonstrating that the global market for traditional medicine continues to be strong. The international market for herbal medicines has hit over $60 billion yearly and it continues to increase gradually [4]. Therefore, medicinal plants such as *Capparis spinosa* (*C. spinosa*) continue to play a major role in healthcare systems [4].

*C. spinosa* is one of the most important economical species in the *Capparidaceae* family which has a wide range of diversity (i.e., about 40–50 genera and 700–900 species) [5]. *Capparidaceae* has been known to be closely related to the family of the *Brassicaceae* (*Cruciferae*) that is rich in glucosinolates and flavonoids [5]. *C. spinosa* is also known as Caper, wild watermelon (in China) [6], Cappero (in Italy), Alaf-e-Mar (in Persian) [7] and Alcapparo (in Spain) [8]. *C. spinosa* is a dicotyledonous perennial shrub which can grow up to 1 m high and has extensive root systems [9]. It is native to the Mediterranean basin and widely distributed from Morroco to Crimea, Armernia, Iran [10]. Several countries such as Greece, Italy, Spain and Turkey have widely produced *C. spinosa* [11]. For example, Spain and Turkey produce about 1000 and 4500 tonnes of *C. spinosa* a year, respectively [8].

Few articles [12,13] have reviewed some of the chemical compounds and health benefits of *C. spinosa* in different aspects including its potential for sustainability. Therefore, the aim of our work was to summarise and review the phytochemical of *C. spinosa* including aerial parts, roots and seeds with reference to their pharmacological activity shown in animal and human studies. In addition, our work has also added significantly to the current understanding of the bioactive compounds isolated from the roots and seeds of *C. spinosa*, which has not been adequately covered in the previous literature reviews.

### 1.1. Search Strategy

An electronic literature search was conducted using Cochrane Library, PubMed, Medline (OvidSP) and Google Scholar until October 2017. Additional references were hand-searched. Search terms included combinations of the following using Boolean markers: *Capparis spinosa*, *C. spinosa*, *Capparis*, caper, phytochemical, pharmacological and antioxidant. The search was restricted to English language publications that addressed the phytochemical constituents and/or pharmacological properties of *C. spinosa*.

### 1.2. Taxonomic Revision of Genus Capparis

There are 813 plant name records that matched *Capparis* in the plant database ‘The Plant List’ (at http://www.theplantlist.org). When *C. spinosa* was searched in the same database, 22 plant name records were found. There have been 250 different species in the genus of *Capparis* which are recognized morphologically. However, the use of morphological markers of the *Capparis* species has its limitations in defining the subspecies and varieties because of the free hybridization of different *Capparis* species and the presence of their intermediate forms [14]. Therefore, the classification of *Capparis* remains to be very ambiguous and controversial [15]. Aichi-Yousfi et al. [16] demonstrated that the use of amplified fragment length polymorphism (AFLP) analysis was efficient to make definitive discrimination among the genetic diversity and relationship between various species of *Capparis*. The authors used three primer combinations of AFLP markers to genotype 213 *Capparis* accessions belonging to six Tunisian *Capparis* species [16].

### 1.3. Cultivation of C. spinosa

*C. spinosa* which is an aromatic plant is usually cultivated in tropical and subtropical regions [10]. The most common propagation of *C. spinosa* is vegetative cuttings [10]. It can flourish under dry hot conditions in either well-drained or poor soils. In addition, *C. spinosa* is salt tolerant and resistant to drought [17]. Although *C. spinosa* can be grown in a wide range of environmental conditions, it is generally grown on sandy loam soils with low alkalinity [18]. It grows and flowers from May to October covering the summer drought [19]. Since it has deep, extensive root systems and can be grown in harsh environments, it has been recommended for the prevention of land degradation and soil erosion control [17].

### 1.4. Traditional Uses of C. spinosa

Different parts of *C. spinosa* including fruits and roots have been used as a traditional herbal remedy since ancient times for its beneficial effects on human diseases [20]. Ancient Egypt and Arab consumed the roots of *C. spinosa* to treat liver and kidney diseases; Ancient Romans used *C. spinosa* for the treatment of paralysis; Moroccans used *C. spinosa* to treat diabetes [8]. In the Northern areas of Pakistan, the root barks of *C. spinosa* have been used to treat splenomegaly, mental disorders and tubercular glands [21]. In China, *C. spinosa* has been used in traditional Uighur Medicine for the treatment of rheumatoid arthritis and gout [6]. In Iran, *C. spinosa* is used to treat hemorrhoids and gout [22]. Table 1 shows the main traditional uses of *C. spinosa* used to ease symptoms and treat diseases.

## 2. Phytochemical Properties of *C. spinosa*

Since *C. spinosa* has several beneficial health effects on human diseases, the chemical and bioactive components of *C. spinosa* have been extensively investigated and reported by numerous analytical studies [11,23,24,25,26,27,28,29,30,31,32,33,34,35,36]. Table 2 demonstrates the important constituents isolated from *C. spinosa*. These studies have focused on the bioactive compounds of different parts of *C. spinosa* such as leaves [27,37], buds [11], fruits [23,24,25,26], roots [31,32,33] and seeds [34,35,36]. In general, *C. spinosa* has a wide range of bioactive compounds such as alkaloids, flavonoids, streroids, terpenoids and tocopherols [38].

Since *C. spinosa* is rich in flavonoids, many studies have identified and quantified flavonoids found in *C. spinosa*. Flavonoid compounds such as rutin and quercertin are detected in *C. spinosa* [39]. Flavonoids have received considerable attention for their positive effects on health because of their anti-oxidative property [40]. Flavonoids are hydroxylated phenolic compounds and are found in plants [39]. Flavonoids have a 15-carbon skeleton, which consists of two benzene rings and a heterocyclic pyrane ring [41]. Flavonoids can be divided into several classes based on their structures such as flavones, flavonols, flavanones, isoflavonoids and others [41]. The consumption of flavonoids is suggested to reduce the risk of cardiovascular disease (CVD) and promote human health [41]. Rutin strengthens capillaries and inhibit platelet clump formation in the blood vessels due to its high radical scavenging and antioxidant properties [42]. In addition, rutin reduces low density lipoprotein (LDL) cholesterol level, which is associated with improve CVD risk biomarkers [43]. Quercetin has been linked with a reduced risk of CVD because of its anti-hypertensive and anti-platelet aggregating properties [44].

### 2.1. Aerial Parts

Most of the isolation and identification of alkaloids and related compounds have been focused on the fruits of *C. spinosa*. A study by Fu et al. [23] reported that the fruits of *C. spinosa* were shown to contain Cappariloside A, stachydrin, hypoxanthine and uracil. Another study by Çaliş et al. [25] reported that two glucose-containing 1*H*-indole-3-acetonitrile compo*unds,* 1*H*-indole-3-acetonitrile 4-*O*-β-(6′-*O*-β-glucopyranosyl)-glucopyranoside and 1*H*-indole-3-acetonitrile 4-*O*-β-glucopyranoside were identified from mature fruits of *C. spinosa* using spectroscopic methods. In addition, capparine A, capparine B, flazin, guanosine, 1*H*-indole-3-carboxaldehyde, 4-hydroxy-1*H*-indole-3-carboxaldehyde apigenin, kaempferol and thevetiaflavone were identified in the fruits of *C. spinosa* [26]*.* There are also other new identified alkaloids including capparisine A, capparisine B, capparisine C that were also isolated from the fruits of *C. spinosa* [45]. Tetrahydroquinoline acid was isolated from the fruits and stems of *C. spinosa* using column chromatorgraphy [46].

Rodrigo et al. [28] reported that fresh *C. spinosa* were shown to contain rutin, kaempferol-3-glucoside, kaempferol-3 rutinoside and kaempferol-3-rhamnorutinoside. Of these flavonoids, rutin was the most abundant [28]. Ramezani et al. [27] reported that the content of rutin in fruits, flowers and leaves of *C. spinosa* was 6.03, 43.72 and 61.09 mg/100 g of dried powder, respectively. It is suggested that rutin may be beneficial to health including cardio-protective versatile, cholesterol-lowering, anti-cancer and anti-inflammatory.

A study by Siracusa et al. [29] reported that rutin, kaempferol 3-*O*-rutinoside and isorhamnetin 3-*O*-rutinoside were isolated from wild-grown *C. spinosa* using High-Performance Liquid Chromatography/Ultraviolet–Visible-Diode Array Spectroscopy/Electrospray Ionization-Mass Spectrometry (HPLC/UV-Vis-DAD/ESI-MS). The authors reported that about half of the total amount of phenolic compounds was rutin (quercetin-3-*O*-rutinoside) followed by kaempferol 3-*O*-rutinoside [29].

In addition, other flavonoids such as quercetin 3-*O*-glucoside, quercetin 3-*O*-glucoside-7-*O*-rhamnoside, quercetin 3-*O*-[6′′′-α-l-rhamnosyl-6′′-β-d-glucosyl]-β-d-glucoside were also identified in the aerial parts of *C. spinosa* [30]. Another study by Zhou et al. [47] reported that ginkgetin, isoginkgetin, sakuranetin, kaempferol-3-*O*-rutinoside and quercetin-3-*O*-rutinoside were isolated in fruits of *C. spinosa*. The authors also reported that ginkgetin, isoginkgetin and sakuranetin were the first time to be identified in fruits of *C. spinosa* [47]. It is suggested that ginkgetin and isoginkgetin may have anti-inflammatory and neuroprotective effects. A study by Sharaf et al. [48] demonstrated that kaempferol-3-rutinoside, quercetin-3-rutinoside, quercetin-7-rutinoside, quercetin 3-glucoside-7-rhamnoside were isolated in stems and leaves of *C. spinosa*.

Afsharypuor et al. [31] reported that leaves and ripe fruits of *C. spinosa* contained the glucosinolates degradation products which were methyl, isopropyl and set-butyl isothiocyanates. Glucosinolates are secondary plant metabolites that are of pharmacological interest because they may have a role in the prevention of diseases and reducing the risk of carcinogenesis. Similarly, Çaliş et al. [25] also reported that indole-3 acetonitrile glycosides was detected in fruits of *C. spinosa.*

Some studies also investigated the flavonoids in flower buds of *C. spinosa* [11,49]. Inocencio et al. [11] used HPLC coupled with a diode-array detector to isolate kaempferol 3-rhamnosyl-rutinoside, kaempferol 3-rutinoside and quercetin 3-rutinoside from the flower buds of *C. spinosa*. The authors reported that rutin (quercetin-3-*O*-rutinoside) was the most abundant compound followed by kaempferol 3-rutinoside [11]. Also, they found that 10 g of *C. spinosa* would provide approximately 65 mg of flavonoid glycosides [11]. The main glucosinolates in shoots and buds of *C. spinosa* was glucocapparin, which contributed 90% of the total glucosinolates [49]. It is suggested that the composition of glucosinolates may vary according to the bud size of *C. spinosa.*

Other classes of compounds such as benzofuranone enantiomers 2-(4-hydroxy-2-oxo-2,3-dihydrobenzofuran-3-yl)acetonitrile were also isolated from the stems and fruits of *C. spinosa* using column chromatography [46]. Two new (6S)-hydroxy-3-oxo-α-ionol glucosides and a prenyl glucoside were isolated from the mature fruits of *C. spinosa* using chromatographic methods [24]. In addition, *p*-hydroxybenzoic acid, vanillic acid, protocatechuric acid, butanedioic acid, uracil, uridine and daucosterol were isolated from fruit of of *C. spinosa* using chromatographic methods [50]. The *p*-methoxy benzoic acid was isolated and identified from the aerial parts of *C. spinosa* using Nuclear Magnetic Resonance (NMR) spectroscopy [51].

In a study investigating the spontaneous volatile emission of different aerial parts of *C. spinosa*, Ascrizzi et al. [52] reported that 178 volatile organic compounds (VOCs) were determined by headspace solid-phase microextraction (HS-SPME) coupled with gas chromatography (GC)/MS. The major VOCs emitted by leaves of *C. spinosa* were germacrene D (20%) and decanal (15%) [52]. The flower bud of *C. spinosa* mainly emitted isopropyl tetradecanoate (26%), (*E*)-nerolidol (17%) and hexahydrofarnesyl acetone (11%) [52]. For the fruits of *C. spinosa*, the majority of the VOCs were β-caryophyllene (40%), α-guaiene (12%), bicyclogermacrene (11%) and macrene B (8%) [52].

### 2.2. Roots

Limited studies have been focused on the isolation and identification of bioactive compounds from the roots of *C. spinosa*. Similar to leaves and fruits of *C. spinosa*, the roots of *C. spinosa* were shown to contain glucosinolates degradation products including methyl, isopropyl and set-butyl isothiocyanates [31]. Fu et al. [53] isolated several new spermidine alkaloids (i.e., capparispine, cadabicine 26-*O*-β-d-glucoside and capparispine 26-*O*-β-d-glucoside) from the roots of *C. spinosa* and used NMR spectroscopy to establish their structures. Stachydrine [32] and 3-hydroxy-7-methoxy-2-methyl-4*H*-1,4-benzoxazine-4-carbaldehyde [33] were also determined from the roots of *C. spinosa* using proton (^1^H) NMR.

### 2.3. Seeds

Ascrizzi et al. [52] reported that the seeds of *C. spinosa* emitted 99% of the total emission profile were sesquiterpene hydrocarbons with the most abundant compounds being β-caryophyllene (45%) followed by α-guaiene (15%), bicyclogermacrene (12%) and germacrene B (8%).

Similar to roots of *C. spinosa*, less attention has been devoted to the seeds of *C. spinosa*. The total content of glucosinolates in seeds of *C. spinosa* (dry weight basis) ranged from 42.6 to 88.9 μmol/g and more than 95% of the total amount of glucosinolates was glucocapperin [36], which is similar to main glucosinolates in shoots and buds of *C. spinosa*. The seeds of *C. spinosa* are also a potential important source of oils for industrial, nutritional and pharmaceutical purposes. The oil content of *C. spinosa* seeds ranged from 27 to 38 g/100 g [36]. Three studies [34,35,36] have shown that the seeds of *C. spinosa* contained two major unsaturated fatty acids, linoleic acid (25–51%) and oleic acid (15–37%), which can improve cardiovascular health. In addition, the seed oils of *C. spinosa* also contain other fatty acids such as myristic acid (1%), stearic acid (3%), palmitic acid (12%), palmitoleic acid (4%), *cis*-vaccenic acid (19%), linolenic acid (1%), behenic acid (1%), eicosenoic acid (<1%), eicosanoic acid (1%) and lignoceric acid (<1%) [36].

Sterols such as stigmasterol, sitosterol, campesterol and avenasterol were also determined from the seed oils of *C. spinosa* [36]. The total amount of sterols in the seed oils of *C. spinosa* ranged from 4962 to 10,008 mg/kg. In addition, the seed oils of *C. spinosa* are also rich in tocopherols with γ- and δ- tocopherols as the major vitamin E active compounds [36]. The total amount of tocopherols in the seed oils of *C. spinosa* ranged from 249 to 1985 mg/100 g [36]. Citrostadienol, cycloartanol, gramisterol, hexadecanol, octadecanol, β-amyrin and tetracosanol were also determined from the seeds of *C. spinosa* [36].

## 3. Pharmacological Effects of *C. spinosa*

Although many studies using various parts of *C. spinosa* have reported diverse pharmacological activities including anti-diabetic and anti-hypertensive, there is still no conclusive information regarding the association between *C. spinosa* and its health benefits. This is because only a very few studies that involved human subjects examined the effect of *C. spinosa* consumption on human health. Similar to other plants [54,55], although there exists literature related to the health benefits of *C. spinosa*, studies often infer a causal correlation between a bioactive substance of *C. spinosa*, and the observe health outcomes. This approach is likely to be an oversimplification of the complex mechanisms in the body that will eventually lead to the observed health outcomes. Therefore, the conclusions of such studies on the effects of changes in the dietary intake should be interpreted with caution because the health outcomes may not be attributed to the action of a single bioactive substance [56]. Table 3 illustrates major pharmacological effect of *C. spinosa*.

### 3.1. Anti-Diabetic

In a randomised, double-blind, placebo-controlled clinical trial of 54 type 2 diabetic patients, Huseini et al. [59] reported that patients who took 1200 mg *C. spinosa* fruit extracts daily for 2 months had a significantly lower level of glycosylated hemoglobin and fasting blood glucose than the control group (*p* = 0.043 and 0.037, respectively). The findings of their study demonstrated an improvement in hypertriglyceridemia and hyperglycemia in diabetic patients [59]. In addition, no renal and hepatic adverse events were reported in the patients [59]. The possible mechanism is that *C. spinosa* decreases the rate of carbohydrate absorption and exert its postprandial hypoglycemic effect in the gastrointestinal tract [58]. Therefore, the consumption of *C. spinosa* may be beneficial and safe for controlling and treating blood glucose levels in diabetic patients.

### 3.2. Anti-Obesity

Eddouks et al. [57] demonstrated that there was a significant weight loss in diabetic rats fed with the aqueous extract of powdered fruits of *C. spinosa* (20 mg/kg) after 2 weeks (*p* < 0.01). Another study by Lemhadri et al. [58] reported that repeated oral administration of aqueous extracts of *C. spinosa* was associated with a significant loss of body weight in high fat diet-fed mice after 2 weeks (*p* < 0.01). Therefore, it is suggested that *C. spinosa* may be used for weight loss management. However, further studies are needed to confirm such findings.

### 3.3. Cholesterol-Lowering

*C. spinosa* has been reported to be associated with improved plasma lipid parameters. Huseini et al. [59] reported a significant decrease in the triglyceride level of type 2 diabetic patients who were supplemented with 1200 mg *C. spinosa* fruit extracts daily for 2 months (*p* = 0.029). The possible mechanism for the cholesterol-lowering effect of *C. spinosa* is that *C. spinosa* decreases the activity of 3-hydroxy-3-methyl-glutaryl coenzyme A reductase (HMG-CoA reductase), which plays an important role in the biosynthesis of cholesterol [69]. Therefore, *C. spinosa* may be useful for the treatment of fatty liver disease and metabolic syndrome because it plays an important role in the inhibition of gluconeogenesis in liver.

### 3.4. Anti-Hypertensive

*C. spinosa* has the potential to be used for the treatment of hypertension. In a study of spontaneously hypertensive rats, Ali et al. [61] reported that when the aqueous extract of powdered fruits of *C. spinosa* (150 mg/kg) was administered for 20 days, the systolic blood pressure was significantly decreased after 8 days (*p* < 0.05), 12 days (*p* < 0.01) and 16 days (*p* < 0.001). In addition, there was also a significant increase in the concentration of urinary sodium (*p* < 0.001), potassium (*p* < 0.001) and chloride (*p* < 0.001) after 20 days [61]. No change in heart rate was observed during the period. In addition, there was also no difference in the activity of plasma angiotensin-converting enzyme (ACE) and renin after 20 days [61]. It is suggested that *C. spinosa* decreases blood pressure by increasing the excretion of renal electrolytes and inhibiting the ACE activity. The inhibition of ACE activity is associated with a decrease in blood pressure [70]. Therefore, *C. spinosa* may play an important in the reduction of blood pressure.

### 3.5. Anti-Microbial

In a study investigating the antibacterial activity of *C. spinosa*, Boga et al. [33] reported that the growth rate of *Deinococcus radiophilus* (*D. radiophilus*) was significantly inhibited by the addition of aqueous extracts from roots of *C. spinosa* as compared to the control. However, no inhibition was shown on the growth rate of *Escherichia coli* (*E. coli*) by the addition of aqueous extracts from roots of *C. spinosa* [33].

Another study by Mahboubi and Mahboubi [22] evaluated the antimicrobial activity of aqueous, ethanolic, ethyl acetate and methanolic extracts of *C. spinosa* roots and fruits. The authors reported that the aqueous extracts of *C. spinosa* roots exhibited a higher antimicrobial activity against a wide range of microorganisms than that of fruit aqueous extracts of *C. spinosa* fruits [22]. The authors demonstrated that the aqueous extracts from roots of *C. spinosa* possessed inhibitory effect against bacteria and fungi including *Staphylococcus aureus* (*S. aureus*), *Staphylococcus saprophyticus* (*S. saprophyticus*), *Staphylococcus epidermidis* (*S. epidermidis*), *Streptococcus pyogenes* (*S. pyogenes*), *Streptococcus mutans* (*S. mutans*), *E. coli*, *Salmonella typhimurium* (*S. typhimurium*), *Shigella dysenteriae* (*S. dysenteriae*), *Shigella flexneri* (*S. flexneri*), *Klebsiella pneumoniae* (*K. pneumoniae*), *Bacillus subtilis* (*B. subtilis*), *Bacillus cereus* (*B. cereus*), *Candida albicans* (*C. albicans*), *Candida glabrata* (*C. glabrata*), *Aspergillus flavus* (*A. flavus*), *Aspergillus parasiticus* (*A. parasiticus*) and *Aspergillus niger* (*A. niger*) [22].

Gull et al. [62] reported that the methanolic extracts of *C. spinosa* stem barks and shoots had the greatest antibacterial activity against *B. subtilis* with growth inhibition zones of 26.8 mm and 24.6 mm, respectively. While the methanolic extracts of *C. spinosa* fruits had the highest growth inhibition zones (24.9 mm) agains*t Pasteurella multocida* (*P. multocida*) followed by *B. subtilis* (23.9 mm), *E. coli* (20.9 mm) and *S. aureus* (17.7 mm) [62]. On the other hand, the highest antibacterial activity of the methanolic extracts of *C. spinosa* flowers and roots was observed against *E. coli* with growth inhibition zones of 26.5 mm and 23.9 mm, respectively [62]. In addition, polysaccharides of *C. spinosa* leaves have been suggested to exhibit antimicrobial activity. Mazarei et al. [71] reported that polysaccharides of *C. spinosa* leaves had a higher antimicrobial activity against *E. coli*, *S. dysenteriae* and *Salmonella typhi (S. typhi).*

Mahasneh [72] reported that butanol extract of *C. spinosa* showed a moderate to good antifungal activity against *C. albicans* and *A. flavus*. In addition, ethanol extracts of *C. spinosa* exhibited weak cytotoxicity against *Helicobactor pylori* (*H. pylori*) isolates [63]. The infection of *H. pylori* is associated with several gastroduodenal diseases such as gastric cancer [73]. It is suggested that the method of preparing extracts and the type of solvent used may affect the microbial activity of *C. spinosa*.

### 3.6. Anti-Inflammatory

In an in vivo mouse model, El Azhary et al. [64] reported that Swiss albino mice treated with *C. spinosa* leaf extracts had a significantly reduced edema than control, demonstrating the anti-inflammatory activity of *C. spinosa*. In addition, the authors also found that *C. spinosa* had significantly decreased the dermis thickness and immune cell infiltration in the inflammatory site. Similarly, Moutia et al. [65] reported that *C. spinosa* leaf extract was shown to exhibit anti-inflammatory activity in vitro on human peripheral blood mononuclear cells (PBMC) obtained from healthy subjects. The authors also found that PBMC treated with the aqueous fraction of *C. spinosa* leaf extract had a significant increase in interleukin (IL)-4 gene expression (an anti-inflammatory cytokine) and a significant decrease in IL-17 gene expression (pro-inflammatory cytokine) [65]. Therefore, these studies suggested that *C. spinosa* leaf extracts exhibit anti-inflammatory activity by inhibiting the pro-inflammatory cytokines expression and immune cell infiltration [64,65].

*C. spinosa* root extracts were reported to relieve pain in complete Freund’s adjuvant (CFA)-induced rheumatoid arthritis and mono-iodoacetate (MIA)-induced osteoarthritis male Sprague-Dawley rats [66]. It is suggested that the pain reliever effect of *C. spinosa* roots could be due to the presence of spermidine alkaloids which have anti-inflammatory effects [66]. Similarly, the lyophilised methanolic extracts from flowering buds of *C. spinosa* was also reported to exhibit anti-inflammatory effect by reducing the production of reactive oxygen species (ROS), nitric oxide (NO) and prostaglandins (PGE_2_) induced by IL-1β on human chondrocytes [74]. These findings were consistent with another study by Feng et al. [75] who demonstrated that the fraction eluted by ethanol-water (50:50, *v*/*v*) from *C. spinosa* fruits was shown to exhibit the most significant anti-arthritic response in male Wistar rats, providing additional evidence that *C. spinosa* possesses anti-inflammatory effects.

Similarly, *C. spinosa* exhibited anti-inflammatory activity to suppress cytokine production of dendritic cells (DC), induced by lipopolysaccharide (LPS). In a study investigating the effect of *C. spinosa* fruit ethanol extracts on the maturation of mouse bone marrow-derived DC Hamuti et al. [67] reported that different types of *C. spinosa* extracts exhibited different effects on the maturation of DC. DC is the key regulator in many aspects in homeostasis of immune system [76] and inflammatory skin disorders [77]. These different types of *C. spinosa* extracts were labelled as *C. spinosa* extract (CSE) 2 water (W), CSE middle-layered isolated (M) W, CSE3W, CSE2 dimethyl sulfoxide (D), CSEMD and CSE3D according to their methods of preparation which were either dissolving in water or dimethyl sulfoxide (DMSO) at a concentration of 200 mg/mL. The authors reported that CSEMD and CSEMW dose-dependently increased the expression of cluster of differentiation (CD) 40, CD80 and CD86 [67]. CSE2W was shown to significantly increase the expression of CD40 but not CD80 [67]. Although CSE3D and CSE3W had significantly inhibited the secretions of pro-inflammatory cytokines including IL-1β, IL-12p40, IL-6 and tumor necrosis factor (TNF)-γ induced by LPS, CSE3D had a higher inhibitory activity than CSE3W [67].

### 3.7. Antihepatotoxic

A study by Gadgoli and Mishra [51] evaluated the effects of *C. spinosa* on the antihepatotoxic on rats against paracetamol and carbontetrachloride induced toxicity in vivo. In addition, the authors also investigated the effects of *C. spinosa* on galactosamine and thioacetamide induced toxicities in vitro [51]. In their study, methanol soluble fraction of the aqueous extract of aerial parts of *C. spinosa* was reported to exhibit significant reductions in serum glutamyl pyruvate transaminase (SGPT), serum glutamyl oxalacetate transaminase (SGOT), alkaline phosphatase and total bilirubin in paracetamol and carbontetrachloride induced hepatotoxicity in albino rats of Wistar strain [51]. In addition, the authors also reported that aerial parts of *C. spinosa* showed significant anti-hepatotoxic activity in galactosamine and thioacetamide induced hepatotoxicity in isolated rat hepatocytes [51].

Kazemian et al. [68] reported that both groups of diabetic rats receiving 0.2 g/kg and 0.4 g/kg of hydroalcoholic extract of *C. spinosa* had a significant reduction of alanine aminotransferase (ALT) and alkaline phosphatase (ALP) (*p* < 0.05 for both groups) after 4 weeks of treatment. Therefore, it is suggested that *C. spinosa* extract does not cause any toxic effect on the liver [68].

### 3.8. Other Pharmacological Properties

*C. spinosa* also exhibits potential protective effects against cognitive impairment. In a study investigating the effect of *C. spinosa* on the learning and memory damage induced by chronic administration of LPS (175 μg/kg) in young male Sprague-Dawley rats, Goel et al. [78] reported that the aqueous extract of *C. spinosa* buds had significantly reduced the neurodegeneration in the first region in the hippocampal circuit (CA1) region of hippocampus, suggesting that *C. spinosa* could be used to treat cognitive disorders.

In addition, another study by Mohebali et al. [79] reported that the expression of β-secretase enzyme (BACE)-1, presenilin protein (PSEN)-1 and PSEN-2 and amyloid precursor protein (APP) genes were significantly down regulated in amyloidogenic related genes in amyloid-beta (Aβ) peptide-injected Wister albino rats treated with hydroalcoholic extracts of *C. spinosa* leaves and fruits as compared to the control group. Another study by Turgut et al. [80] reported that the extracts of *C. spinosa* seeds significantly protected against the damage of DNA bands and attenuated cognitive impairment induced by d-galactose in male, Bagg albino, laboratory-bred strain of the House Mouse (BALB/c) mice. Chronic neurodegenerative diseases such as Alzheimer’s disease are associated with the aggregation and misfolding of proteins. The accumulation of Aβ peptide is the key protein involved in the development of Alzheimer’s disease and the accumulation of Aβ peptide can be caused by malfunction in β- and γ- secretase enzymes. BACE is encoded by BACE-1; γ-secretase enzyme is encoded by PSEN-1 and PSEN-2 [81].

*C. spinosa* also exhibits inhibitory effects on human immunodeficiency virus (HIV)-1 reverse transcriptase [82]. Lam and Ng [82] isolated a protein with an N-terminal amino acid sequence from the seeds of *C. spinosa* that inhibited HIV-1 reverse transcriptase. The protein exhibited some similarly to imidazoleglycerol phosphate synthase [82]. Lam and Ng [82] reported that the protein possessed HIV-1 reverse transcriptase inhibitory activity with the half maximal inhibitory concentration (IC_50_) of 0.23 μM. It is suggested that the inhibition is due to the protein-protein interaction which causes homologous retroviral reverse transcriptase to be inhibited by HIV-protease [82].

### 3.9. Adverse Effects

Currently, the consumption of *C. spinosa* is not associated with any adverse effects according to the published literature, providing evidence that *C. spinosa* is safe to consume [83].

## 4. Conclusions

A literature review has highlighted that *C. spinosa* exhibits important pharmacological effects because *C. spinosa* is rich in many bioactive compounds including flavonoids. Therefore, given the few clinical studies cited, it is very risky to highlight the potential role of *C. spinosa* on treatment such as diabetes, hypertension and obesity. This suggests that there are many opportunities for the food and healthcare industry to explore the health benefits of *C. spinosa* because there is a potential growth market for *C. spinosa*. However, the majority of the studies that reported beneficial effects of *C. spinosa* on health are animal-based studies. Moreover, these studies used different parts of *C. spinosa* plant, types of solvents and methods of preparation, which cause the evaluation of activity of *C. spinosa* difficult. In addition, these studies involve quite heterogeneous data.

Therefore, future prospective research should screen for individual polyphenol constituents that possess health-promoting properties in *C. spinosa*. This is because a cause-effect relationship between *C. spinosa* and its health effects can only be established when the composition of *C. spinosa* is properly characterised and standardised. In addition, there is limited evidence of randomized controlled trials on humans to support such health beneficial effects of *C. spinosa* when compared to other plants such as walnuts that have high economic interests to the food industry. The underlying mechanism influencing human health by the consumption of *C. spinosa* still remains unclear. The effect of short- and long-term consumption of *C. spinosa* on human health therefore needs to be further evaluated. Furthermore, the role of the gut microbiota in the degradation of polyphenolic compounds present in the plant has not been considered. This aspect should be put forward in the perspectives [84].

## Figures and Tables

**Table 1:** Main traditional uses of *Capparis spinosa* (*C. spinosa*) to ease symptoms and treat diseases.

Treated Symptoms and Diseases
Toothache
Fever
Headache
Menstruation
Rheumatism
Convulsions
Gout
Skin disease
Kidney disease
Liver disease
Diabetes
Hemorhoids
Ulcers
Sciatica

**Table 2:** Summary of some important constituents isolated from *C. spinosa*.

Parts of *C. spinosa* Plant	Compounds Identified	Extraction Method	References
Aerial parts	Cappariloside A	Chromatographic method	Fu et al. (2007) [23]
	Stachydrin		
	Hypoxanthine		
	Uracil		
	1*H*-indole-3-acetonitrile 4-*O*-β-(6′-*O*-β-glucopyranosyl)-glucopyranoside	Spectroscopic method	Çaliş et al. (1999) [25]
	1*H*-indole-3-acetonitrile 4-*O*-β-glucopyranoside		
	Indole-3 acetonitrile glycosides		
	Capparine A	Spectroscopic method	Zhou et al. (2010) [26]
	Capparine B		
	Flazin		
	Guanosine		
	1*H*-indole-3-carboxaldehyde		
	4-hydroxy-1*H*-indole-3-carboxaldehyde		
	Apigenin		
	Kaempferol		
	Thevetiaflavone		
	Capparisine A	Chromatographic method	Yang et al. (2010) [45]
	Capparisine B		
	Capparisine C		
	Tetrahydroquinoline	Chromatographic method	Zhang et al. (2014) [46]
	Benzofuranone enantiomers 2-(4-hydroxy-2-oxo-2,3-dihydrobenzofuran-3-yl)acetonitrile		
	Rutin	Chromatographic method	Mollica et al. (2017) [37]
	Kaempferol-3-glucoside	Chromatographic method	Rodrigo et al. (1992) [28]
	Kaempferol-3 rutinoside		
	Kaempferol-3-rhamnorutinoside		
	Kaempferol 3-*O*-rutinoside	Chromatographic method	Siracusa et al. (2011) [29]
	Isorhamnetin 3-*O*-rutinoside		
	Quercetin 3-*O*-glucoside	Chromatographic method	Sharaf et al. (2000) [30]
	Quercetin 3-*O*-glucoside-7-*O*-rhamnoside		
	Quercetin 3-*O*-[6′′′-α-l-rhamnosyl-6′′-β-d-glucosyl]-β-d-glucoside		
	Kaempferol 3-rhamnosyl-rutinoside	Chromatographic method	Inocencio et al. (2000) [11]
	Kaempferol 3-rutinoside		
	Quercetin 3-rutinoside		
	Ginkgetin	Spectroscopic method	Zhou et al. (2011) [47]
	Isoginkgetin		
	Sakuranetin		
	Quercetin-3-*O*-rutinoside		
	Quercetin-7-rutinoside	Chromatographic method	Sharaf et al. (1997) [48]
	Glucocapparin	Chromatographic method	Matthaus & Ozcan (2002) [49]
Roots	Capparispine	Spectroscopic method	Fu et al. (2008) [27]
	Cadabicine 26-*O*-β-d-glucoside		
	Capparispine 26-*O*-β-d-glucoside		
	Stachydrine	Chromatographic method	Khatib et al. (2016) [32]
	3-hydroxy-7-methoxy-2-methyl-4*H*-1,4-benzoxazine-4-carbaldehyde	Chromatographic method	Boga et al. (2011) [33]
Seeds	Glucocapparin	Chromatographic method	Matthaus & Ozcan (2002) [49]

**Table 3:** Overview on major pharmacological effects of *C. Spinosa*.

Pharmalogical Effects	Models	Parts of *C. spinosa* Plant Used	References
Anti-diabetic	Streptozotocin-induced diabetic rats	Fruits	Eddouks et al. (2005) [57]
	Highly glucose tolerant and high fat diet-fed mice	Fruits	Lemhadri et al. (2007) [58]
	Type 2 diabetic patients	Fruits	Huseini et al. (2013) [59]
	Streptozotocin-induced diabetic rats	Leaves	Mollica et al. (2017) [37]
Anti-obesity	Streptozotocin-induced diabetic rats	Fruits	Eddouks et al. (2005) [57]
	Highly glucose tolerant and high fat diet-fed mice	Fruits	Lemhadri et al. (2007) [58]
Cholesterol-lowering	Streptozotocin-induced diabetic rats	Fruits	Eddouks et al. (2005) [57]
	Streptozotocin-induced diabetic rats	Fruits	Jalali et al. (2016) [60]
	Type 2 diabetic patients	Fruits	Huseini et al. (2013) [59]
Anti-hypertensive	Spontaneously hypertensive rats	Fruits	Ali et al. (2007) [61]
Antimicrobial	Cell culture	Roots	Boga et al. (2011) [33]
	Cell culture	Roots and fruits	Mahboubi & Mahboubi (2014) [22]
	Cell culture	Stem barks and shoots	Gull et al. (2015) [62]
	Cell culture	Aerial parts	Masadeh et al. (2014) [63]
Anti-inflammatory	Swiss albino mice	Leaves	El Azhary et al. (2017) [64]
	Human peripheral blood mononuclear cells	Leaves	Moutia et al. (2016) [65]
	Male Sprague-Dawley rats	Roots	Maresca et al. (2016) [66]
	Mouse-bone marrow derived dendritic cells	Fruits	Hamuti et al. (2017) [67]
Antihepatotoxic	Albino rats of Wistar strain	Aerial parts	Gadgoli & Mishra (1999) [51]
	Diabetic rats		Kazemian et al. (2015) [68]
